# Evaluation of Clinical Manifestations of Hemorrhoidal Disease, Carried Out Surgeries and Prolapsed Anorectal Tissues: Associations with ABO Blood Groups of Patients

**DOI:** 10.3390/jcm12155119

**Published:** 2023-08-04

**Authors:** Inese Fišere, Valērija Groma, Šimons Svirskis, Estere Strautmane, Andris Gardovskis

**Affiliations:** 1 Department of Doctoral Studies, Rīga Stradiņš University, Dzirciema Street 16, LV-1007 Riga, Latvia; 2 Surgery Clinic, Pauls Stradins Clinical University Hospital, Pilsonu Street 13, LV-1002 Riga, Latvia; andris.gardovskis@rsu.lv; 3 Institute of Anatomy and Anthropology, Rīga Stradiņš University, Dzirciema Street 16, LV-1007 Riga, Latvia; 4 Institute of Microbiology and Virology, Rīga Stradiņš University, Ratsupītes Street 5, LV-1067 Riga, Latvia; ssvirskis@latnet.lv; 5 Medical Faculty, Rīga Stradiņš University, Dzirciema Street 16, LV-1007 Riga, Latvia; esterestrautmene99@gmail.com

**Keywords:** hemorrhoidal disease, clinical manifestations, surgery, prolapsed anorectal tissue, ABO blood groups, morphology

## Abstract

Hemorrhoidal disease (HD) is a chronic multifactorial disease. Increased abdominal pressure, along with hyperperfusion, neovascularization, overexpression of inflammatory mediators, and dysbiosis, contributes to the development of HD. The deterioration of the anchoring connective tissue with reduced collagen content and altered collagen ratios, dilatation of blood vessels and thrombosis, muscle injury, and inflammation gradually lead to clinically manifesting prolapse and bleeding from hemorrhoids. The associations of the ABO blood types with a disease have been investigated for the upper gastrointestinal tract only. This study aimed to evaluate HD clinical manifestations, surgeries carried out, and the status of prolapsed anorectal tissues by exploring the associations with the patients’ ABO blood groups. Clinical and various morphological methods, combined with extensive bioinformatics, were used. The blood type 0, grade III and IV HD individuals constituted the largest group in a moderately-sized cohort of equally represented males and females studied and submitted to surgical treatment of hemorrhoids. There were significantly more complaints reported by HD females compared to males (*p* = 0.0094). The Longo technique appeared mostly used, and there were proportionally more surgeries performed below the dentate line for HD individuals with blood type 0 compared to other blood type patients (24% vs. 11%). HD males were found to present with significantly more often inflamed rectal mucosa (*p* < 0.05). Loosening and weakening of collagenous components of the rectal wall combined with vascular dilation and hemorrhage was found to differ in 0 blood type HD individuals compared to other types. HD males were demonstrated to develop the ruptures of vascular beds significantly more often when compared to HD females (*p* = 0.0165). Furthermore, 0 blood type HD males were significantly more often affected by a disease manifested with tissue hemorrhage compared to the 0 blood type HD females (*p* = 0.0081). Collectively, the local status of chronically injured anorectal tissue should be considered when applying surgical techniques. Future studies could include patients with HD grades I and II to gain a comprehensive understanding of the disease progression, allowing for a comparison of tissue changes at different disease stages.

## 1. Introduction

Hemorrhoidal disease (HD) is one of the most common anorectal diseases, affecting 13–36% of the general population [1,2,3,4,5,6,7,8]. The prevalence of HD is estimated as high as 88%, depending on the definition used [9,10]. About 50% of people over the age of 50 years have complaints due to HD [1,4,11,12]. Identical in both genders, the disease commonly affects individuals aged 45 to 65 [1,4,11,12,13,14,15,16,17,18], while external thrombosed hemorrhoids are reported at a younger age (<45) and are more pronounced in women [12,19].

The most studied and generally accepted risk factors for HD are chronic constipation and slow bowel movements [4,11,12,15,20,21]. However, several additional risk factors—aging, obesity, straining, pregnancy, lifestyle, spicy food, alcohol, sedentary lifestyle, and physical activity are mentioned [7,15,20,22,23]. Low fiber intake (<12 g/day) and insufficient hydration (<2 L/day) are reported to increase the risk of HD development [22]. Reversely, sufficient use of a fiber-rich diet is demonstrated to reduce HD symptoms and bleeding by 50% [24]. Advancing age and strenuous lifting, straining with defecation, and prolonged sitting are believed to contribute sufficiently to the abnormal downward displacement of the anal cushions causing venous dilatation in HD [1,3,4,25]. Pathophysiological studies highlight increased abdominal pressure as a significant factor [2,11,25,26], along with hyperperfusion, neovascularization [7,27], overexpression of inflammatory mediators [21], and dysbiosis [28]. Histopathological changes include the deterioration of anchoring connective tissue with reduced collagen content and altered collagen ratios [24,29,30,31]. All these factors collectively weaken the perivascular connective tissue and lead to the dilation of blood vessels, resulting in the formation of hemorrhoids. 

HD has very different symptoms and manifestations—itching, swelling, anal discharge, hygienic problems, bleeding, and pain [2]. A total of 40% of individuals with HD are asymptomatic [1,3,4,14,32]. For internal hemorrhoids, the most commonly reported symptom is bleeding and the sensation of tissue prolapse [1,6], whereas the most frequently reported patient complaints are dyschezia, constipation, and diarrhea [2,6,13,14,15,21]. 

Surgery is recommended for high-graded internal hemorrhoids, failed non-operative approaches, or HD complications [1,2,4,11,12,13,14,26,33]. Commonly, only 5 to 10% of patients require surgical hemorrhoidectomy [2,3,4,11,12,13,34]. Minimally invasive procedures, such as LigaSure hemorrhoidectomy, Doppler-guided hemorrhoidal artery ligation, and stapled hemorrhoidopexy have been introduced as alternatives to excisional hemorrhoidectomy. However, it is still used for patients presented with complications [1,4,11,21,25,26,35]. Both the conventional Milligan–Morgan (MM) and closed Ferguson and Parks hemorrhoidectomy techniques have similar complications: tissue trauma, pain, bleeding, mucosal discharge, prolonged local care, and anal stricture [13,25,26]. The Ferguson technique shows better results in terms of wound healing, postoperative pain, and bleeding [8,16,19,36]. The Longo technique preserves hemorrhoidal tissue by relocating and anchoring prolapsed internal hemorrhoids and anoderm [1,4,11,13,14,25]. Therefore, the use of stapled hemorrhoidopexy has some rationale behind it [9,37]. Stapled hemorrhoidopexy addresses mucosal prolapse, but traditional stapled hemorrhoidopexy may cause unnecessary tissue damage [6,38]. A higher hemorrhoidopexy staple line is shown to demonstrate better functional results, with a lower risk of incorporating part of the internal sphincter and the anal transitional zone [39,40,41].

A link between the ABO blood group system and disease has been demonstrated [32,42,43,44,45]. The ABO blood group system, consisting of three main alleles, is controlled by a single gene located on chromosome 9. The association between ABO blood type and bleeding risk and thromboembolic diseases is influenced primarily by the plasma levels and biologic activity of the Von Willebrand factor (VWF) and glycosyltransferase activity [8,23,24,46,47,48]. In the case of VWF, glycosylation is essential for its proper folding, multimerization, and stability in circulation. In turn, alterations in glycosylation can lead to structural and functional changes in VWF, affecting its ability to mediate platelet adhesion and clot formation. On the other hand, the ABO blood group system is reported to affect some specific aspects of platelet function as well [12] found that blood group 0 is a potential genetic risk factor for bleeding, while [29] found that blood group 0 is associated with increased bleeding severity in patients with bleeding of unknown cause. Moreover, blood group 0 has been associated with an increased risk of severe hemorrhages and lower hemostatic potency observed in upper gastrointestinal bleeding [23,28,29,30]. For the upper gastrointestinal tract, oral mucosal bleeding, peptic ulcer disease, erosive disease, and cirrhosis-associated gastroesophageal variceal bleeding are recognized as the most common causes of bleeding [31]. Although some evidence suggests an association between ABO blood type and bleeding from hemorrhoids, most often confirmed in patients with blood group 0 and the least common in B and AB blood group individuals [44,49], current understanding of the relationship between the development of hemorrhoids and different ABO blood groups is incomplete. Therefore, several research questions appeared to be addressed in the given study, as follows: (i) what are the clinical manifestations of HD in patients of different ABO blood groups? (ii) How does the local status of chronically injured anorectal tissue influence the choice and success of surgical techniques for HD? (iii) Are there significant differences in reported complaints between male and female HD patients, particularly related to ABO blood types? (iv) How do the composition and condition of collagenous components of the rectal wall and anal canal differ in HD patients with blood type 0 compared to other blood types? (v) Are there any gender differences among HD patients with blood type 0 experiencing hemorrhage? (vi) Are there differences in the presence of chronically injured anorectal tissue and inflamed rectal mucosa between male and female HD patients? To address the aforementioned issues, we conducted this study to evaluate HD clinical manifestations, surgeries carried out, and the status of prolapsed anorectal tissues by exploring the associations with patients’ ABO blood groups. In pursuit of this goal, we also attempted the assessment of possible differences between HD in younger and older male and female patients.

## 2. Materials and Methods

### 2.1. Patients’ Characteristics

In this observational study, the study population was chosen from the database of 316 patients who were treated surgically from September 2020 to June 2021 at Pauls Stradins Clinical University Hospital, Riga, Latvia. The database used for patient selection was comprehensive and included HD patients from regional medical centers, thus enhancing the representativeness of the study population. Sixty adult, equally distributed male and female patients clinically presented with HD grades III and IV were found eligible for inclusion in the study. The recruited participants were representative of the target population, ensuring external validity. Inclusion criteria were defined as follows: age over 18 years, HD grades III and IV, refractory to conservative measures, and persistent clinical manifestation for the last year. Exclusion criteria were concomitant anorectal inflammatory disease (fistula, abscess, inflammatory bowel disease), colon or anal cancer, history of colon or anorectal operation, additional or multiple surgeries required, and active use of immunosuppressants. Patients underwent a thorough anamnesis and physical examination, including inspection, digital-rectal examination, and ano/rectoscopy. The hemorrhoidal disease was defined as symptomatic according to the Goligher classification. All patients underwent hemorrhoidectomy or hemorrhoidopexy surgery. Post-interventional follow-up included clinical examination and ano/rectoscopy after four weeks. Medical records and anorectal tissues of these patients were used in this retrospective study. The certified proctologist collected the data, thus ensuring accuracy and consistency. The information on gender, age, blood group, complaints, type of operation, complications, and data from the histopathological examination was extracted from the record and assessed for each patient. Only standardized data collection protocols and measurement tools were used to minimize observer bias and ensure objectivity and consistency in data gathering. The sum of reported complaints was scored as follows: 1—one complaint recorded; 2—two complaints recorded; 3—three complaints recorded; and 4—four different complaints recorded. The study was approved by the Ethical Committee of Riga Stradins University (Decision No. 22-2/264/2021) and conducted according to the Declaration of Helsinki. Any sensitive information was excluded, and the necessity for informed consent was waived due to the retrospective design of the study.

### 2.2. Histopathological and Histochemical Investigation of Anorectal Tissues

Sixty surgically obtained formalin-fixed and paraffin-embedded (FFPE) anorectal tissue samples were sectioned and mounted on SuperFrost Plus slides (Gerhard Menzel GmbH, Braunschweig, Germany). Routine histopathological staining with hematoxylin and eosin was performed to confirm the diagnosis of HD and accurately detect the site either above or below the dentate (pectinate) line and surfaced by simple columnar or stratified squamous epithelium, accordingly. Van Gieson’s and Picro Sirius red staining were used for the assessment of collagen. The sections stained in Picro Sirius red were further observed under interference and polarized light that improves the visualization and assessment of collagen fibers using a Biolar microscope (BIOLAR, Warsaw, Poland) [30,31,49]. The density of connective tissue fibers appearing in Van Gieson’s and Picro Sirius red staining was graded semiquantitatively from 0 to 4, where: 0—loose, 1—minimally dense, 2—moderately dense, 3—markedly dense, and 4—very dense. Furthermore, in each of the slides, the presence of inflammatory cells, various blood vessels, and hemorrhage was assessed. The structures were estimated in 5 properly oriented microscopic fields for each region of interest. The sections were examined by a Leitz light microscope (LEICA, LEITZ DMRB, Wetzlar, Germany) using a digital camera DC 300F, whereas the images of interest were captured using a Glissando Slide Scanner (Objective Imaging Ltd., Cambridge, UK).

### 2.3. Immunohistochemical Investigation of Anorectal Tissues

The expression of vascular type V collagen and CD34 was assessed immunohistochemically. For this purpose, 4–5 µm-thick FFPE sections mounted on SuperFrost Plus slides (Gerhard Menzel GmbH, Braunschweig, Germany) were used. The IHC protocol recommended by the manufacturer was applied. Briefly, deparaffinized sections were incubated overnight with the primary mouse monoclonal anti-collagen type V antibody (Abcam, Cambridge, UK, 1:300 dilution, ab201980) and mouse monoclonal anti-CD34 antibody (Invitrogen, Camarillo, CA, USA, 1:200 dilution, BI-3C5) at 4 °C. A HiDef Detection HRP Polymer system and a diaminobenzidine tetrahydrochloride substrate kit (Cell Marque, Rocklin, CA, USA) were used to visualize the products of IHC reactions. Cell nuclei were counterstained with Mayer’s hematoxylin. Primary antibodies were omitted in the negative controls of IHC reactions. The reaction results were assessed by two independent observers blind to the clinical data.

### 2.4. Statistical Data Analysis

Statistical data analysis, as well as graphing, were performed using Prism 9 software for macOS (GraphPad Software, LLC, San Diego, CA, USA) and JMP 16 (SAS, Cary, NC, USA). To improve the comparability of patient groups and increase the reliability of associations between ABO blood groups and clinical manifestations, a propensity score matching (PSM) test was performed using the XLSTAT 2023.1.6 (1410) package for Mac as a preliminary analysis. Clinical parameters were presented as medians with an interquartile range (IQR). The histopathology and histochemistry data and immunostaining values within the group were analyzed using the Wilcoxon signed ranks. The Chi-squared test was applied to compare the distribution of variables used to characterize the extent of inflammation in HD tissue samples of males and females. Spearman’s rank correlation analysis was applied to detect the possible correlation between patients’ complaints and histopathology data, as well as the relation between sex, age, type of surgery, and ABO blood type. A hierarchical clustering method was used to explore the similarities and differences of data, and to see a pattern of data obtained, whereas alluvial plots were constructed to show how associations across categorical dimensions of variables are allocated. The difference between variables was considered significant at *p* < 0.05.

## 3. Results

### 3.1. General Clinical Information

**Figure 1:**
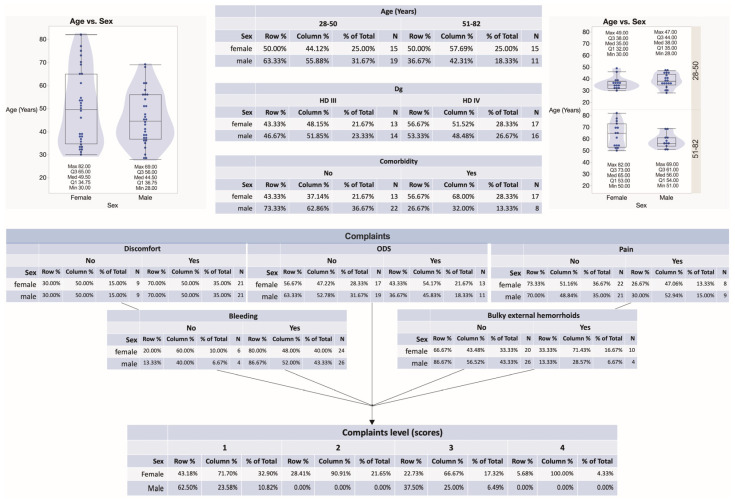
A summary of patients’ characteristics related to age, sex, HD staging, complaints, and comorbidities.

**Figure 2:**
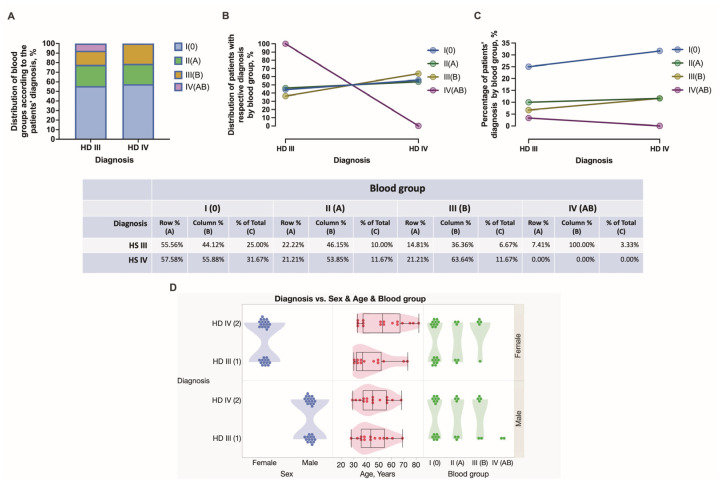
Distribution of female and male patients of different ages presented with HD grade III and IV according to their blood group data. The distribution of blood groups according to diagnosis (**A**). Distribution of patients with respective diagnosis by blood group (**B**). Percentage of patient diagnosis by blood group (**C**). Diagnosis vs. sex and age and blood groups (**D**).

**Figure 3:**
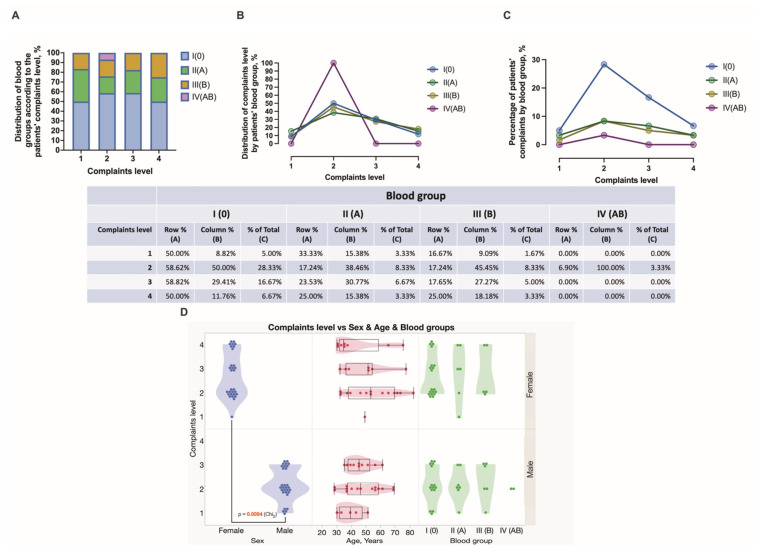
A spectrum and distribution of complaints reported in HD patients with different blood groups. Distribution of blood groups according to the complaints level (**A**–**C**). Distribution of complaints level vs. sex and age and blood groups (**D**).

During the study time span, 60 subjects were diagnosed with HD stage III and IV and further submitted to hemorrhoidectomy surgery and anorectal tissue retrieval. The patients’ age, sex, HD staging, complaints, comorbidities, and stratification into four blood groups are summarized in Figure 1, Figure 2 and Figure 3. Among all recruited patients, 13 (21.67%) female patients and 14 (23.33%) male patients were diagnosed with HD stage III, whereas 17 (28.33%) female patients and 16 (26.67%) male patients were diagnosed with HD stage IV (Figure 2). In the present study, a major portion of patients presented by blood group 0. There were 55.56, 22.22, 14.81, and 7.41% of HD grade III patients presented by blood groups 0, A, B, and AB, respectively. Simultaneously, there were 57.58, 21.21, and 21.21% of HD grade IV patients presented by blood groups 0, A, and B, respectively. Among HD grade IV patients, no one presented by blood group AB. Two HD grade III males presented by blood group AB (Figure 2). There were no significant differences found between males and females presented by different blood groups. Equal numbers of females were presented in a group of patients aged from 28 to 50 years and in a group of patients aged from 51 to 82 years. In turn, 19 (31.67%) males constituted a group of patients aged under 50 years, whereas 11 (18.33%) males constituted a group of patients aged above 51 years (Figure 1). There were no significant differences found between males and females under and above 50 years, as well as males and females diagnosed with HD stage III and stage IV (Figure 2).

### 3.2. Comorbidities, HD Patients’ Complaints, and Types of Surgery Used

**Figure 4:**
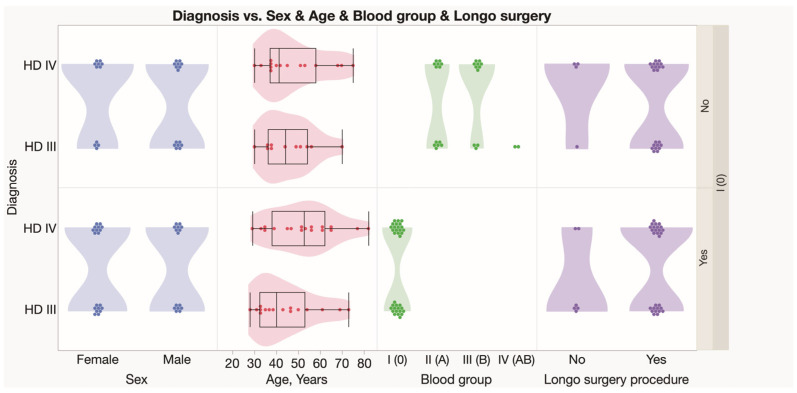
Type of surgery carried out on patients of grade III and IV HD with different blood groups.

There were seventeen females and seven males presented with comorbidities, whereas 13 (21.67%) females and 22 (36.67%) males did not reveal any comorbidity. Nearly the same number of female and male subjects reported patient complaints. Hemorrhoidal bleeding, anal and perianal discomfort, and ODS were the most common complaints reported. In this study, nine males and eight females reported pain, whereas four males and ten females reported the presence of bulky external hemorrhoids (Figure 1). Specifically, all individuals with blood group A reported only one complaint. In this study, blood group 0 individuals reported several complaints most often. Males had two or three complaints, whereas females had two, three, or more complaints. There were significantly more complaints reported by HD females compared to males (*p* = 0.0094). Simultaneously, no differences were found between males and females in different age groups (Figure 3D). The Longo technique appeared mostly used for HD individuals with blood type 0 compared to other blood type patients (24% vs. 11%) (Figure 4).

### 3.3. Histopathological Stratification and Assessment of Tissue Samples Localized above and below the Dentate Line

**Figure 5:**
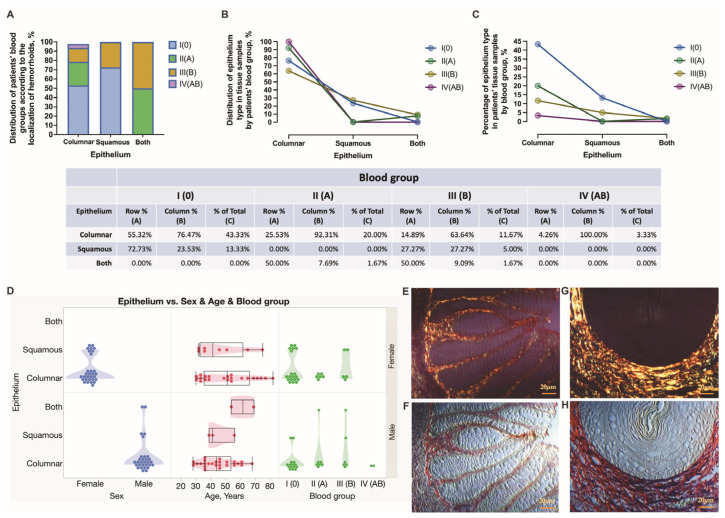
This figure demonstrates a pattern of the histopathological stratification of tissue samples localized above and below the dentate line in HD males and females presented with different blood types (**A**–**D**). Elongated and paralleled crypts that are embedded in the loose connective tissue can be viewed in a polarized light (**E**) and a differential interference mode (**F**). A stratified squamous epithelium that rests on the basement membrane is followed by tightly packed collagenous bundles supporting the basement membrane and is assessed using a polarized light (**G**) and a differential interference mode (**H**). When observed under polarized light, the lamina propria mucosae reveals type I and type III collagen fibers stained in yellow-red and green, respectively. Picro Sirius red staining (**E**–**H**). Scale bars: 20 μm.

Sixty surgically obtained tissue samples of grade III and IV HD cases were used in this study. Histopathologically, the tissues were stratified into those showing either a simple columnar or stratified squamous epithelium as a lining of a mucous membrane and those that included both types joined by the transition zone, and, therefore, reflecting the tissues of internal and external hemorrhoids, respectively (Figure 5). Differently shaped crypts were embedded in the loose connective tissue of the lamina propria mucosae (Figure 5E,F), whereas a stratified squamous epithelium was found to be resting on more tightly packed connective tissue bundles that supported the basement membrane (Figure 5G,H). The HD 0 blood group individuals constituted a larger group of patients whose surgical samples were obtained above the dentate line, followed by A, B, and AB blood group individuals—55.32, 25.53. 14.89, and 4.26%, respectively. In turn, the presence of stratified squamous epithelium was confirmed in 72.73, 0.00, 27.27, and 0.00% of 0, A, B, and AB blood group individuals, respectively (Figure 5A). Finally, the surgical samples across the transition zone with both types of epithelia accounted for 50% of A and B blood group individuals only (Figure 5A–C). Females presented with surgical samples below the dentate line more often than males. In turn, two males only presented with tissue samples across the transitional zone (Figure 5D).

### 3.4. Assessment of Chronic Anorectal Tissue Injury in Hemorrhoidal Disease Patients

**Figure 6:**
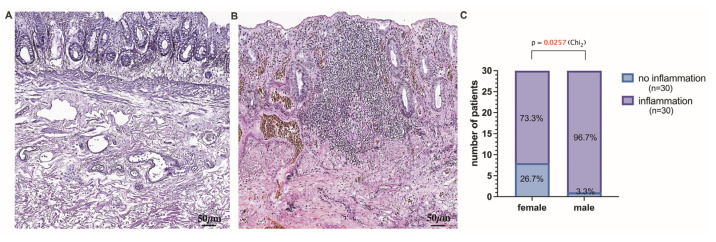
A representative image that depicts the histopathological features of mucosa and submucosa in HD in the absence (**A**) and presence (**B**) of inflammation. (**C**) The extent of inflammation in the anorectal samples obtained from males is significantly higher than that in females. (**A**) The rectal mucosa reveals paralleled crypts; however, the glands do not rest on the muscular mucosae; the submucosa houses thin- and thick-walled vessels. (**B**) The culprit bleeding vessels are visualized in the rectal mucosa. Inflammatory infiltrate brings to the reduction and distortion of crypts and produces insertions toward the haphazardly patterned muscular mucosae. Van Gieson’s staining (**A**,**B**). Scale bars: 50 μm.

**Figure 7:**
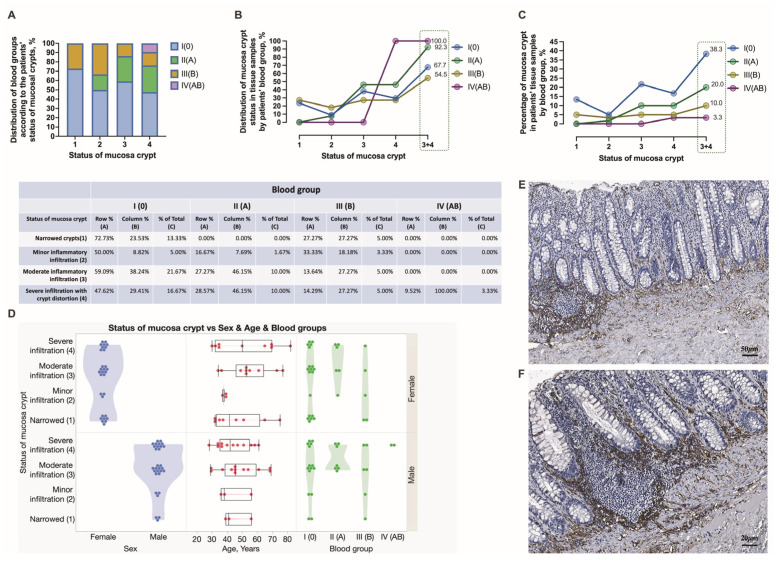
The histopathological assessment of chronic mucosal lesions in HD males and females presented with different blood groups. The schematic presentation of the structural appearance of crypts in HD patients of 0, A, B, and AB blood types (**A**–**C**). The status of mucosal glands is graded and estimated in HD males and females of different age groups (**D**). A representative image that depicts the histopathological features of inflamed mucosa HD (**E**,**F**). CD34 immunohistochemistry (**A**,**B**). Scale bars: 50 μm (**A**), 20 μm (**B**).

**Figure 8:**
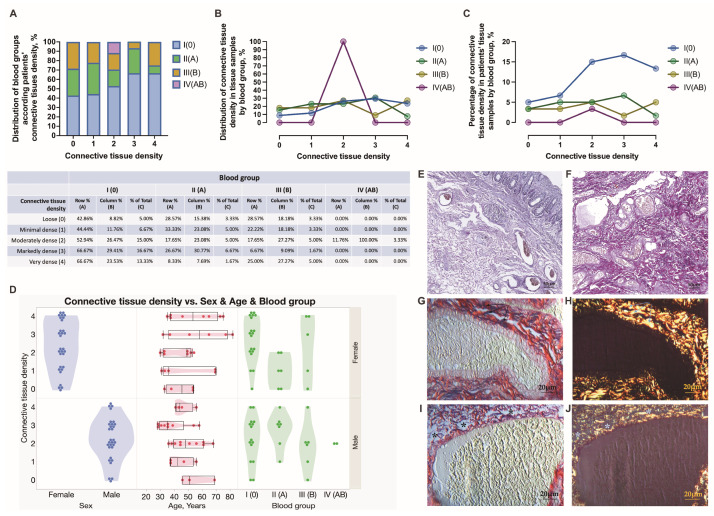
The histochemical assessment of connective tissue collagen fibers in HD males and females presented with different blood groups (**A**–**D**). Loosely (**E**) or more densely (**F**) structured submucosa houses thick and thin-walled congested blood vessels, Van Gieson’s staining (**E**,**F**). Scale bars: 50 μm. Perivascular collagen is assessed in thick (**G**,**H**) and thin-walled (**I**,**J**) vessels using a differential interference (**G**,**I**) and a polarized light mode (**H**,**J**). When observed under polarized light, type I and type III collagen fibers are stained in yellow-red and green, respectively. Picro Sirius red staining (**H**,**J**). Scale bars: 20 μm.

**Figure 9:**
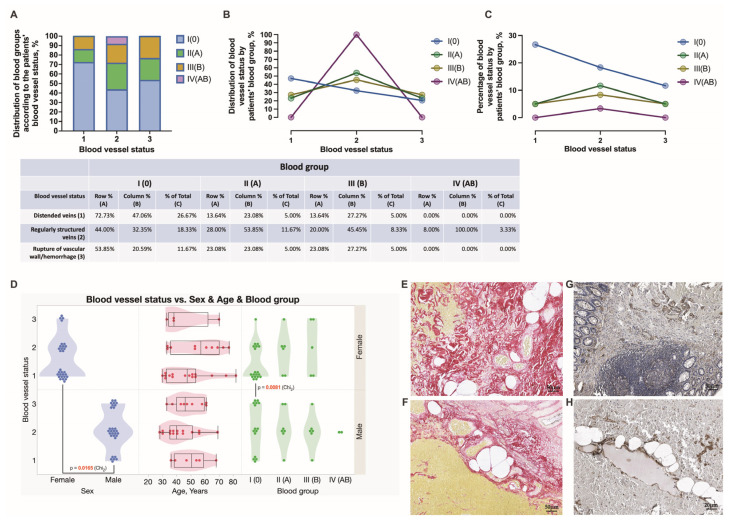
This figure illustrates the architectural peculiarities of collagen and its contribution to the maintenance of vascular integrity. Distribution of blood groups according to the patients blood vessel status (**A**). Distribution of blood vessels status by patients blood group (**B**). Distribution of blood vessel status by patients blood group in percentage (**C**). Blood vessel status vs. sex and age and blood group (**D**). Small arterioles are embedded in a collagenous matrix that reveals a haphazard orientation of fibers partly inserted into the hemorrhage area (**E**). Congested and markedly enlarged submucosal vessels (**F**). Inflamed mucosa with crypt distortion and thickened muscular mucosa; small mucosal and submucosal vessels are labeled with the anti-collagen type V antibody (**G**). Small dilated submucosal vessels labeled with the anti-collagen type V antibody are surrounded by a large hemorrhage (**H**). Picro Sirius red staining (**E**,**F**). Collagen type V immunohistochemistry (**G**,**H**). Scale bars: 50 μm (**E**–**G**), 20 μm (**H**).

Chronic mucosal injury of the rectum, including the presence of inflammatory cells, follicular lymphoid hyperplasia, crypt distortion, and crypt shortfall, was evidenced in the tissue samples obtained from HD patients (Figure 6A,B). In this study, anorectal tissues obtained in males were significantly more often inflamed (96.7% vs. 3.3%) than those in HD females (Figure 6C). In addition, for a better exploration of chronic mucosal lesions in HD, immunostaining was performed to specify the involvement of vascular and immune cells. Specifically, CD34 immunohistochemistry was applied to track the mucosa coat vascularity and the presence and distribution of immune cells contributing to crypt distortion. Endothelial cells of small mucosal blood vessels were positively stained with the anti-CD34 antibody and, therefore, easily tracked and analyzed. As a surface molecule, CD34 was found decorating immunocompetent cells infiltrating mucosa (Figure 7E,F). The mucosa crypt status was assessed extensively, and a degree of crypt damage was scored (Figure 7A–D). Severely lesioned and inflamed mucosa was found in 47.62, 28.57, 14.29, and 9.52% of 0, A, B, and AB blood group individuals, respectively (Figure 7D). Both females and males of 0, A, and B blood groups and males of AB blood type presented with severely lesioned rectal mucosa. The significant differences between females and males and different age groups of HD patients when estimating chronic mucosal lesions were not found. The estimation of anorectal tissues was extended to the submucosa, and the integrity and density of collagen fibers, including perivascular fibers, were explored by the use of special stainings—Van Gieson’s and Picro Sirius’s red stainings (Figure 8E–J) and scored (Figure 8A–C). Loosening of connective tissue collagen was confirmed in HD tissue samples. The presence of loosened collagen arrangement in the tissue samples was confirmed and further assessed proportionally to the representative number of HD subjects studied. It was determined in a large proportion of HD individuals of 0 blood type—42.86%, followed by HD individuals of A and B blood types—28.57 and 28.57%, respectively. No significant differences were found when assessing collagen density in HD males’ and females’ samples and different age groups (Figure 8D). Immunohistochemistry was applied to target the expression and distribution of vascular type V collagen and explore its contribution to the integrity of the vascular wall. The results were assessed using Picro Sirius red staining and type V collagen immunohistochemistry in all anorectal tissue samples (Figure 9E–H). The presence of dilated and ruptured submucosal veins was confirmed in 72.73, 13.64, 13.64, and 0%, and 51.35, 27.03, 18.92, and 2.70% of HD individuals of 0, A, B, and AB blood types, respectively (Figure 9A–C). There were significant differences found when assessing the vascular integrity in HD males’ and females’ samples but not the age groups (Figure 9D). In this study, HD males were demonstrated to develop the ruptures of vascular beds significantly more often when compared to HD females (*p* = 0.0165). Furthermore, 0 blood type HD males were significantly more often affected by a disease manifested with tissue hemorrhage compared to the 0 blood type HD females (*p* = 0.0081).

### 3.5. Hierarchical Clustering Used for the Exploration of Data Similarities and Visualization Using Alluvial Plotting

**Figure 10:**
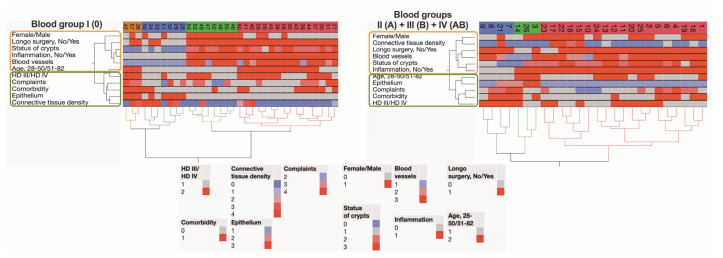
Hierarchical clustering shows the similarities of data points related to the studied system. The clades and leaves of a tree included data on the histopathological evaluations of a variety of anorectal tissue parameters in 60 subjects with grade III and IV HD submitted to surgical treatment, information about a patient’s age and sex, complaints, and comorbidities.

**Figure 11:**
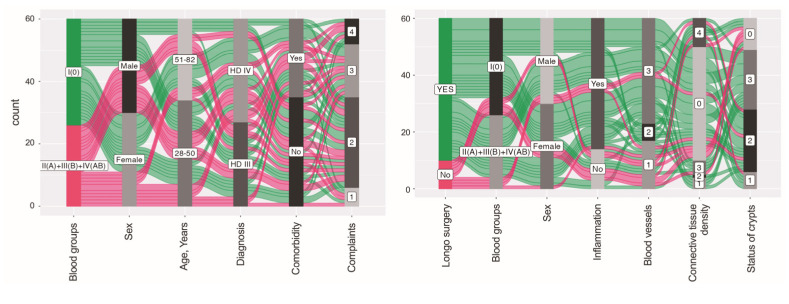
Alluvial diagrams represent flows among nodes. In these diagrams, individual observations are presented as rows, while characteristics are presented as columns. The width of each line and the flow path that stems from it are determined by the proportional fraction of the category total. The left panel illustrates how many patients of 0 blood type/patients of A, B, and AB blood types were males/females aged below or over 50 years, presented with HD grade III/IV, had comorbidities, and reported one or more complaints. The right panel shows how a high proportion of HD females and males have undergone Longo surgery or other types of surgical treatments, had 0 or A, B, and AB blood types, and presented with inflammation, damaged blood vessels and mucosal glands, and a certain density of connective tissue in their samples.

A hierarchical clustering method was used to assess the similarity and differences in the levels of studied factors. Data for patients in the HD 0 blood group were analyzed separately from data of HD subjects of A, B, and AB blood types (Figure 10). There were four different clusters recognized for patients in the group HD 0. A small, orange-colored cluster was distinguished in the HD 0 blood group only. However, some similarities have emerged regarding patient complaints and comorbidities, but not the site of surgery, with a blue cluster representing HD subjects with blood type A, B, and AB. Green cluster consisting only of HD 0 blood group males undergoing Longo surgery presented with an inflamed mucosa, heavily deformed crypts, and ruptured blood vessels revealing hemorrhage. In a red-colored cluster consisting of HD patients of A, B, and AB blood types submitted to Longo surgery, the levels of certain studied factors, specifically, the presence of inflammation, deformation of crypts, and damage of blood vessels, were recognized to resemble those determined for HD 0 blood type individuals. Additionally, alluvial diagrams were plotted to show how associations across categorical dimensions of variables are allocated. This type of data representation was used to better visualize an individual spectrum of the variables studied (HD patients of 0 blood type vs. HD patients of A, B, and AB blood types, and HD patients submitted to Longo surgery vs. HD patients submitted to other types of surgical treatments) and the relationship between variables such as sex, age, HD grade, comorbidity, complaints, submission to Longo surgery, and anorectal tissue-related characteristics (Figure 11).

### 3.6. Multivariate Analysis of Hemorrhoidal Disease Contributing Factors

**Figure 12:**
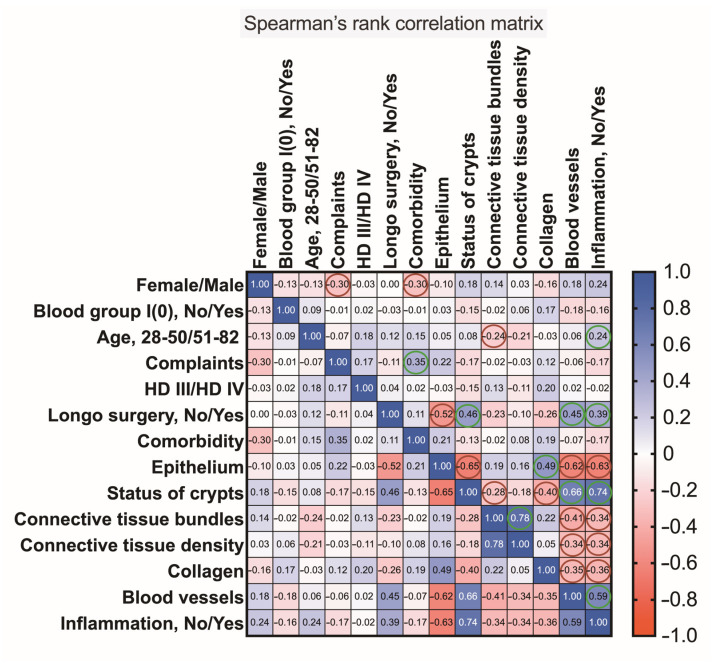
A correlogram of the studied variables. In this graph, correlation coefficients are colored according to the value. Positive correlations are displayed in blue, whereas negative correlations are shown in red. Color intensity is proportional to the correlation coefficients. Colored circles indicate associations with a significance level of *p* < 0.05.

Multivariate analysis was used to better recognize a pattern of data obtained and to investigate the dependence between multiple variables at the same time. A correlation matrix, as plotted and demonstrated in Figure 12, indicates that in this study, females reported complaints slightly more often than males (r = −0.30). Elderly persons with HD were more likely to develop inflammation than younger patients with HD (r = 0.24). As predicted, one patient’s complaints were positively associated with comorbidities (r = 0.35). The application of the Longo technique appeared to be more beneficial for patients presented with a simple columnar epithelium in their tissue samples when compared to those presented with a stratified squamous epithelium or the transitional zone (r = −0.52). This technique was applied to patients who presented with crypt distortion (r = 0.46), hemorrhage (r = 0.45), and inflammation (r = 0.39). Inflammatory infiltrate that surrounded a crypt was determined more frequently when compared to the stratified squamous epithelium (r = −0.63), and hemorrhage was confirmed more often in the mucosa coat covered by a simple columnar epithelium (r = −0.62). A decrease in the density of connective tissue was associated with hemorrhage (r = −0.34) and larger inflammatory infiltrates (r = −0.34). Severely distorted crypts were strongly associated with ruptured blood vessels, hemorrhage, and the presence of larger infiltrates (r = 0.74). Finally, the occurrence of ruptured blood vessels with hemorrhage was confirmed to be moderately associated with tissue inflammation (r = 0.59).

**Figure 13:**
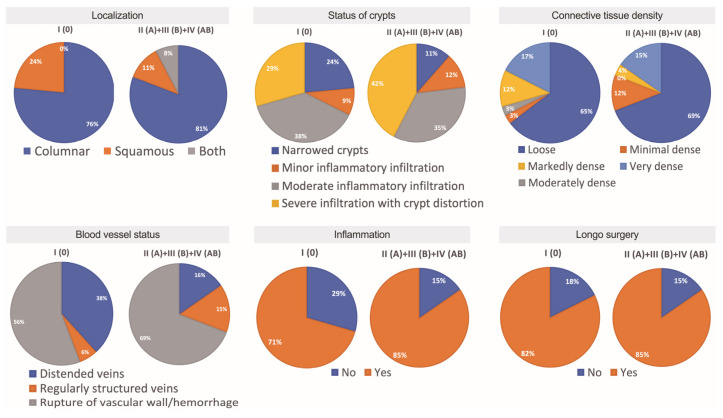
Comparison of the proportion of categorical variables studied for HD individuals of 0 blood type and other blood types.

Finally, a comparative analysis of the proportion of main categorical variables explored in this study for HD patients of blood type 0 and other blood types was performed (Figure 13). There were proportionally more surgeries performed below the dentate line for HD individuals of blood type 0 compared to other blood type patients (24% vs. 11%). Furthermore, the 0 blood type individuals presented more often with normally appearing mucosal glands compared to other blood type patients (24% vs. 11%). Proportions for minimally and markedly densely arranged connective tissue collagen fibers were differently presented in HD individuals of blood type 0 compared to other blood type patients, as it appears in a pie chart. Notably, dilated submucosal veins were two times more common (38% vs. 16%) in the samples of HD individuals with blood type 0 when compared to other blood types. Simultaneously, anorectal tissues obtained from the 0 blood type individuals were less inflamed (29% vs. 15%) when compared to other blood types. Finally, Longo surgery was equally applied in all HD patients.

## 4. Discussion

In this study, HD clinical manifestations, surgeries carried out, and the status of prolapsed anorectal tissues by exploring the associations with patients’ ABO blood groups were analyzed. Younger and older male and female HD patients were investigated to target the possible differences between the aforementioned groups of subjects. To reach the goal, various histopathological methods, microscopies, and extensive bioinformatics tools were used. The given study also has some features of novelty. Firstly, the novelty of this study lies in exploring clinical manifestations and reported complaints among HD patients, particularly concerning their ABO blood types, which adds a novel perspective to understanding the disease’s clinical presentation. Secondly, the association between ABO blood groups and the status of prolapsed anorectal tissues in HD patients has been investigated, thus contributing to a comprehensive understanding of the disease and its potential underlying mechanisms. Thirdly, the novelty lies in investigating the potential gender-specific differences in the presence of inflamed rectal mucosa among HD patients and the predisposition to tissue hemorrhage in HD patients, particularly in relation to blood type 0. Finally, the assessment of the integrity and density of collagen, a bulk of anorectal tissue, and its loosening, contributing to the development of HD, provides a better understanding of the disease’s pathophysiology.

Hemorrhoids are vascular submucosal cushions that prevent stool leakage from the rectum [2,3,13,14,20]. They are classified based on their position relative to the dentate line: internal hemorrhoids above the line, covered by simple columnar epithelium, and external hemorrhoids below the line, covered by stratified squamous epithelium [1,2,21]. Studies have linked various factors, such as aging, genetics, intestinal issues, and dysbiosis, to the development of hemorrhoids [25,28,50]. Among other HD development promoting factors, abnormal connective tissue morphology and dysregulated blood flow in the affected anal cushions have been mentioned [1,2,7,15,18,37]. However, the complex analysis of tissue stratified by the location of the lesion and associated HD surgery has not yet been fully performed. Additionally, no prior research has explored potential connections between clinical symptoms, surgical treatments, and different ABO blood types in individuals with hemorrhoids, making this study novel.

Various surgical methods for hemorrhoid therapy have distinct benefits, indications, contraindications, and complications. However, no gold standard exists. Conventional excisional hemorrhoidectomy is commonly used for grade III and grade IV HD treatment, with energy devices helping to reduce postoperative pain and bleeding [51]. The MM technique involves leaving wounds open for 3–5 weeks for healing. On the other hand, the Longo technique is considered safe and effective for grade II and III HD [2,4,11,13,14,26,27,32], showing excellent short-term results [2,8,16,18,19]. However, its efficacy for larger and more complicated HD treatments, as well as long-term follow-up, remains debated [8,13,19]. Other authors have found that conventional hemorrhoidectomy had more complications and a higher recurrence rate compared to stapled hemorrhoidopexy (Longo) within one year [1,4,32]. Long-term results of the Longo procedure with a 12-year follow-up were also evaluated [1,4,32]. Several systematic reviews suggested that stapled hemorrhoidopexy was less effective than conventional hemorrhoidectomy [1]. In Europe, particularly in France, Italy, and the United Kingdom, the MM-modified technique is widely used [51]. Modified techniques utilizing LigaSure tissue-sealing devices allow for faster operations with reduced bleeding and tissue damage, especially with increasing surgical experience [52]. In our study, stapled hemorrhoidopexy appeared as a surgical technique used most often and with fewer postoperative complications.

Numerous postoperative complications have been reported, including bleeding, relapses, surgical site hematoma, thrombosis, urine retention, incontinence, stenosis, and recto-vaginal fistula in female patients [42,53,54,55,56]. However, the frequency of complications has decreased since 2015 [36]. Postoperative pain after stapled hemorrhoidopexy is associated with various factors such as incorrect technique, anal wounds, inflammation in the stapled ring, age, or pelvic floor nerve stimulation [38,44,54,56,57,58]. Pain after the MM application is linked to bleeding and inflammation, often leading to acute reoperations to stop postoperative bleeding [6]. Delayed post-hemorrhoidectomy bleeding (DPHB) is an important complication that can occur between the fourth and eighteenth days after the procedure [26]. Risk factors for DPHB include the use of the LigaSure device by the surgeon and constipation, with an incidence of 0.9–49% [6]. On the contrary, other studies reported the early period hemorrhage—within three days [25]. In our study, DPHB was established on days 4, 5, 7, and 8 postoperatively when applying the Longo technique, and only once bleeding was demonstrated after LigaSure hemorrhoidectomy. Rates of postoperative bleeding have been reported as 2.4% and 5.7% in patients who underwent stapled procedures and hemorrhoidectomy, respectively [59]. Secondary bleeding after hemorrhoid surgery occurs in 0.6–2.4% of cases, with the highest probability on the sixth to ninth postoperative day [34]. Severe postoperative bleeding is recognized in 0.7% of patients only [60].

Previously, a reduction in the amount of connective tissue collagen was shown in HD. Other authors have reported a decrease in the ratio of type I to III collagen leading to the impaired mechanical stability of the perivascular tissue in HD patients [30,31,49,61]. However, approval of whether changes in collagen formation in HD are caused by external factors and metabolic changes or are influenced by genetic factors remains unsolved. Recently, connective tissue dysfunction was proven in HD-predisposed individuals conducting genome-wide analysis [23]. In the aforementioned study, prioritized genes encoding macromolecules, including collagens highly expressed in hemorrhoidal tissue, were identified. Furthermore, COL5A2, which encodes for type V collagen, was found to be implicated in the development of HD. Type V collagen is widely recognized as an essential molecule for major collagen types I and III fibril formation. The results of the collagen assessment obtained in the given study are in line with these observations. In this study, a decrease in the density of connective tissue was accompanied by gradual dilation of submucosal veins and hemorrhage. Furthermore, loosening and weakening of collagenous components of the rectal wall combined with vascular dilation and hemorrhage was found to differ in 0 blood type HD individuals compared to other types. HD males were demonstrated to develop the ruptures of vascular beds significantly more often when compared to HD females, and 0 blood type HD males were significantly more often presented with tissue hemorrhage compared to females. Other authors suggested that venous dilation and ruptures are the sources of bleeding. Furthermore, vascular malformations may become susceptible to injuries during defecation, and hemostatic failure may gradually develop in HD [47]. The exploration of the vascular supply and blood flow to the hemorrhoidal tissues is of value when developing advanced HD treatment techniques, including hemorrhoidal de-arterialization procedures [46,48,62]. Stapled hemorrhoidopexy lifts the hemorrhoidal cushions back up into their normal anatomical position and interrupts the mucosal and submucosal internal hemorrhoidal vascular pedicles, thus reducing the arterial flow.

HD is characterized by severe vasodilatation, which causes inflammation of the surrounding tissues and leads to extravasation in the interstitial space, increased permeability of blood vessels, fragility, and migration of inflammatory cells [24,63,64]. An increase in vascular permeability in chronically injured and inflamed tissue was shown to occur along with the elevation of the expression of CD34 and other adhesion molecules on the surface of vascular endothelial and immune cells [65].

In this study, we investigated the mucosa coat vascularity and the presence and distribution of immune cells contributing to crypt distortion using CD34 immunohistochemistry. Even rather small mucosal blood vessels were positively stained with the anti-CD34 antibody and easily tracked. The immunocompetent cells infiltrating mucosa were scored, and the contribution of larger inflammatory infiltrates to crypt distortion was estimated. HD males were found to present with significantly more often inflamed rectal mucosa. Elderly patients with HD were reported to develop inflammation more likely than younger patients in connection with the duration of the pathology, ischemia, vasculitis, toxins, medications, chronic tissue injury, diet (citrus, coffee, cola, beer, garlic, spices, and sauces), and smoking [64]. The presence of inflammation in the anorectal passage before the operation can be considered reliable when using preparations containing flavonoids, which are anti-inflammatory, antibacterial, vasoconstrictive, bleeding-reducing, and wound-healing [24,50,63]. Notably, some flavonoids were shown to inhibit gene expression and some pro-inflammatory cytokines [57].

The associations between ABO blood type and disease have been studied since the early 1900s when researchers determined that blood plasma antibodies and antigens on the surface of red blood cells are inherited [30,42,51,66]. The ABO blood group system consists of three main alleles and is controlled by a single gene located on the terminal portion of the long arm of chromosome 9 (9q34. 2) [32,42,43,52]. The link between the ABO blood type and thromboembolic diseases and bleeding risk is intervened by the glycosyltransferase activity and plasma levels, and the biologic activity of Von Willebrand factor, a carrier protein for coagulation factor VII which is low in the blood group 0 [24,27,47,52,53,54]. Previously, an ABO blood group 0 was demonstrated to have an important role in upper gastrointestinal bleeding [30]. The blood group 0 was shown to be associated with an increased risk of developing more severe hemorrhages and lower hemostatic potency [29,42,44,52,55]. Furthermore, blood group 0 was shown to be prevalent in patients with bleeding for unknown reasons compared to the normal population and to be associated with increased bleeding severity similar to bleeding from esophageal, gastric, and duodenal ulcers [29,44,66]. However, the difference was not confirmed when studying the associations between postpartum hemorrhage and blood type [23,53]. Simultaneously, the subjects other than the blood group 0 were reported to have an increased risk of developing coronary heart disease and venous thrombosis compared with 0 blood group individuals [12,44,52]. Therefore, the presence of non-0 blood type somehow may be assumed to be predisposing for thromboses [32,54,56]. Simultaneously, patients with blood group AB were shown to have a significantly higher 90-day survival compared with other blood groups [29,55]. In turn, this investigation proved that individuals with 0 blood type who have hemorrhoids (HD) show differences in the rectal wall’s collagenous components, vascular dilation, and hemorrhage compared to individuals with other blood types. Additionally, HD males with 0 blood type are more prone to developing ruptures of vascular beds and experiencing tissue hemorrhage compared to HD females with 0 blood type.

The results of this study must be viewed in light of some limitations. The relatively small sample size is one of the limitations of the given study. It was performed on a small-sized HD patient cohort. This limitation may affect the generalizability of the findings to a larger population. The authors acknowledge that certain characteristics were assessed mainly through categorical variables. This might limit the depth of analysis and understanding of complex relationships between variables. The study aimed at the anorectal tissue assessment, and the resected tissues were obtained from advanced HD grade III and IV patients. Therefore, the authors did not explore the plausible tissue changes to occur earlier in HD grade I-II patients. Next, due to the retrospective character of the study, some clinical data remained not specified and analyzed in the present study, which might bring some new results and interpretations. Finally, realizing that the 0 blood group individuals constitute the majority worldwide, we created a non-0 group representatives’ group by combining A, B, and AB blood group patients. This grouping might not account for potential differences between individual blood groups. However, a combined group of patients with blood groups A, B, and AB and a group of the 0 blood group individuals contrasting them were equalized, and the proportions of characteristics were assessed.

## 5. Conclusions

This retrospective study provides valuable insights into a spectrum of clinical manifestations and surgical treatments applied to treat prolapsing HD, as well as the assessment of anorectal tissue changes associated with the disease. The observed mucosal inflammation, connective tissue loosening, and vascular wall ruptures leading to hemorrhage are symptoms of prolapsing HD across all blood types—additionally, the presence of multiple complaints increases in patients with comorbidities. The study demonstrates the effectiveness of the most commonly used surgical therapy for curing HD and offers important information on the origin of patients’ complaints, the applied therapy, and the localization of tissue lesions subjected to further resection. There was a higher level of tissue inflammation observed in men. However, further investigation is needed to understand its potential impact on the presentation of local symptoms in the context of edema. Notably, anorectal tissues obtained from HD individuals with blood type 0 showed relatively lower levels of inflammation compared to other blood types, while congested and markedly dilated submucosal veins were more frequently found in HD individuals with blood type 0. Given the relatively small cohort size, future studies should aim for a larger sample to enhance the generalizability of the findings and improve the statistical power for detecting significant associations. To gain a comprehensive understanding of the disease progression, future studies, when possible, should include patients with HD grades I and II, allowing for a comparison of tissue changes at different disease stages. Future studies should carefully account for confounding variables, such as age, gender, and comorbidities, to obtain more robust and reliable findings. Finally, collaborating with multiple medical centers can increase the diversity of the patient population and improve the external validity of the findings.

## Data Availability

A publicly available bibliographic database, PubMed.gov, was used in this study (accessed from May 2020 till July 2023). The complete search query is specified in Section 2 of the article. The full bibliographic reference list is available upon request from the corresponding author.
